# Supplementary material for: Circadian control of brain glymphatic and lymphatic fluid flow
Source: Nat Commun. 2020 Sep 2;11:4411. doi: 10.1038/s41467-020-18115-2 (PMC7468152; doi:10.1038/s41467-020-18115-2)
Supplement: Supplementary file 1 — Supplementary Information [file 41467_2020_18115_MOESM1_ESM.pdf]

Supplementary information for:

“Circadian control of brain glymphatic/lymphatic fluid flow” by Hablitz *et al.*

Supplementary Figures

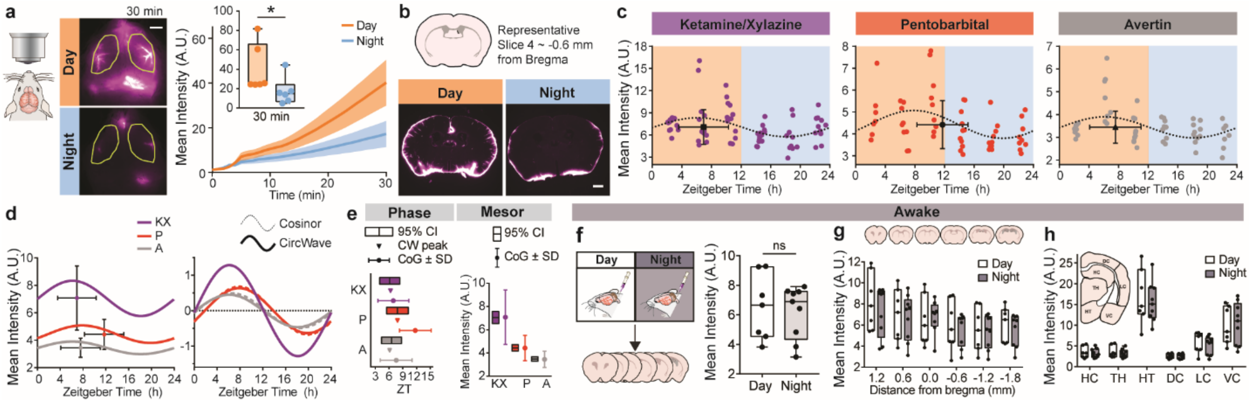

Supplementary Fig. 1. Glymphatic influx has a diurnal rhythm in anesthetized mice. (a) Representative images for transcranial imaging during day (orange) and night (blue) under ketamine/xylazine (KX) anesthesia (left). White scale bar: 2 mm. Solid yellow lines: quantification regions. (right) Mean intensity (arbitrary units (A.U.)) over 30 min. Thick lines: means, shading: SEM. Inset: boxplot of fluorescence at 30 min. Time 0: tracer infusion start. n = 6 mice per group. Mann Whitney test: $p = 0.026$. Asterisk indicates $p < 0.05$. (b) Representative day/night slices under KX anesthesia. White bar: 1 mm. (c) Mean intensity over time under KX (violet, n = ZT22: 10 mice; ZT18: 11 mice; ZT2, ZT6, ZT10, and ZT14: 12 mice), Pentobarbital (red, n = ZT2: 7 mice; ZT6, ZT10, and ZT22: 9 mice; ZT18: 10 mice; ZT14: 11 mice), and Avertin (gray, n = ZT22: 7 mice; ZT2 and ZT6: 8 mice; ZT14: 9 mice; ZT10 and ZT18: 10 mice). Colored points: individual animals. Dotted line: CircWave (CW) curve. Black point with bars: estimated Center of Gravity (CoG) and s.d. from CW. Light cycles: orange (day) or blue (night) background. Zeitgeber Time (ZT) 0 lights on, ZT12 lights off. (d) CW curves and CoG (left) for KX, pentobarbital (P), and avertin (A). Mesor-aligned CW and Cosinor curves (right), dotted lines: cosinor fit, solid lines: CW fit. (e) 95% confidence intervals from cosinor (center line: cosinor estimate, boxes: upper and lower 95% boundaries), CoG estimates from CW (estimate ± SD: dot and bars), peaks from CW curve for phase and mesor. (f) Schematic of experiment, boxplot of mean intensity for awake mice. Day: white, night: gray, ns: not significant. For f-h: day n = 7 mice, night n = 9 mice. (g) Boxplot of mean intensity from anterior to posterior. Slice diagrams above. (h) Boxplot of mean intensity for hippocampus (HC), thalamus (TH), hypothalamus (HT), dorsal, lateral, and ventral cortex (DC, LC, VC, respectively). Inset: schematic of subregions. All boxplots minima: minimum value, maxima: maximum value, center: median, quartiles: box and whiskers, individual animals: colored dots. Source data are provided as a Source Data file.

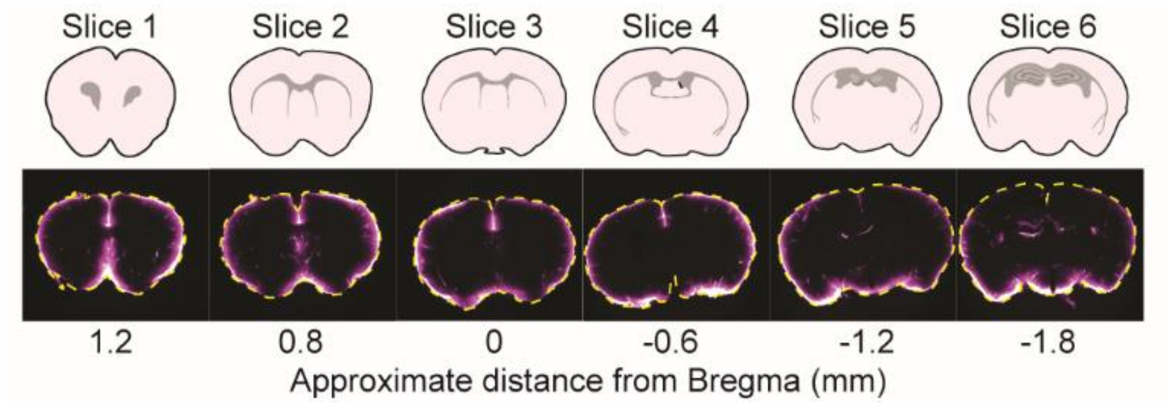

Supplementary Fig. 2. Overview of slice analysis. (Top) Cartoon images of the six slices normally collected after CM injection of tracer. (Middle) Representative images from a brain receiving a CM injection of BSA647. Tracer shown in purple and white, yellow dotted line indicates ROI drawn around slice. (Bottom) Estimated distance from Bregma of each slice.

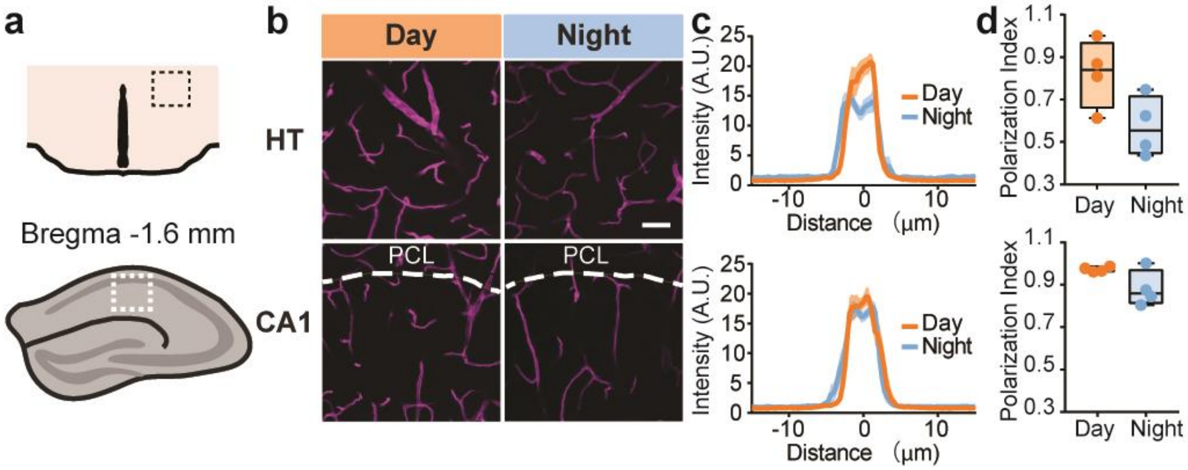

Supplementary Fig. 3. Quantification of AQP4 polarization in Hypothalamus and Hippocampus. (a) Schematics of the hypothalamus (HT) and CA1 of the hippocampus where in the brain images in (b) were taken. Dotted boxes indicate regions of interest for imaging. (b) representative images for HT and CA1 during the day (orange) and night (blue) for AQP4 (magenta) polarization analysis. Dotted lines indicate pyramidal cell layer (PCL) in hippocampus. White scale bar: 50 µm. (c) Average intensity of AQP4 staining centered on vasculature in HT (top) and CA1 (bottom) during the day (orange) and night (blue), with SEM indicated by shading around the thick line representing the group mean. (d) Average polarization index boxplots: minima is minimum value, maxima is maximum value, center is median and quartiles shown by box and whiskers, with individual mice shown as colored dots. Polarization index equals peak vascular end foot fluorescence minus 10 µm baseline. For cd: n = 4 mice, 36 vessels per group. All values were normalized to the highest signal for ease of visualization. Source data are provided as a Source Data file.

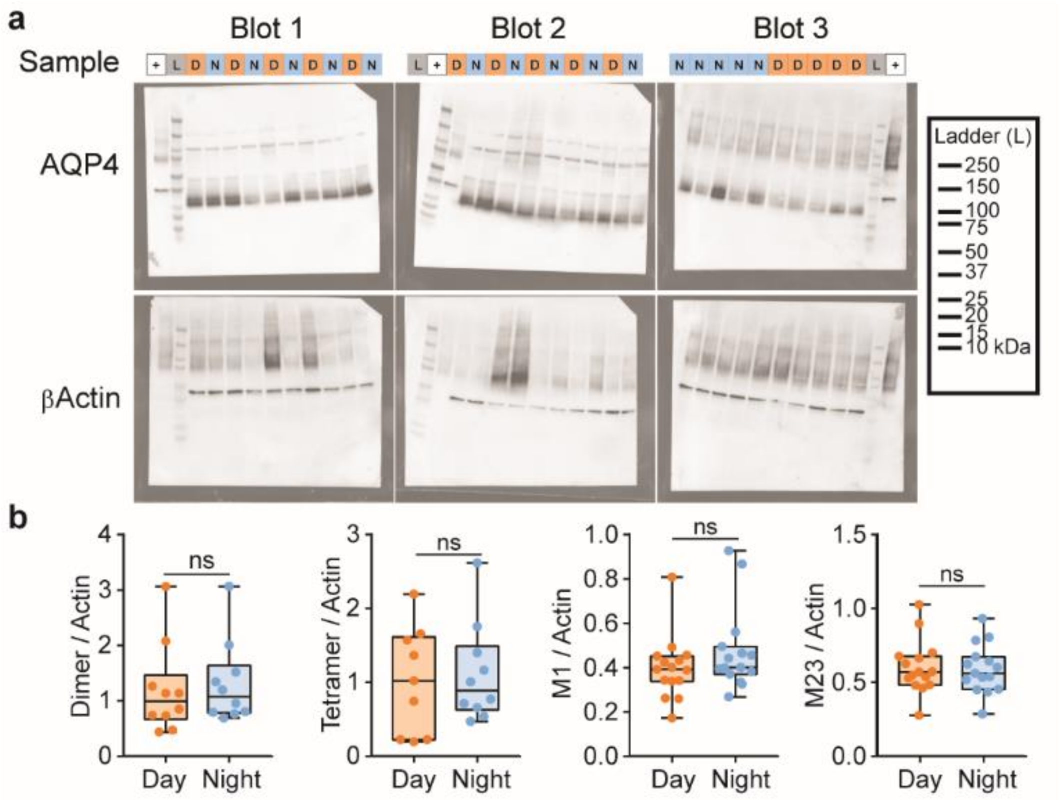

Supplementary Fig. 4. Western blots. (a) Three un-cropped western blots stained with AQP4 primary antibody (top), stripped, and then re-probed for βActin primary antibody (bottom). Sample code is a plus sign for positive antigen control (n = 3 lanes total), L for ladder (n = 3 lanes total), D for day (n = 15 mice), and N for night (n = 15 mice). Blot number is at the top, primary antibody is indicated to the left. On the right is a schematic of the ladder. The antigen control is the rat homolog of AQP4, hence the different molecular weight. AQP4 complexes (dimers and tetramers) are located at higher molecular weights, common for membrane-associated proteins. (b) Boxplots (minima is minimum value, maxima is maximum value, center is median and quartiles shown by box and whiskers) of densitometry for different molecular weight bands of AQP4 normalized to corresponding β-actin loading control. Individual animals are represented by dots. Dimer/Actin: n = 10 mice per group; Tetramer/Actin: n = 9 mice day, 10 mice night; M1/Actin: n = 15 mice per group; M23/Actin: n = 15 mice per group. ns indicates non-significance using a two-tailed t-test. Source data are provided as a Source Data file.

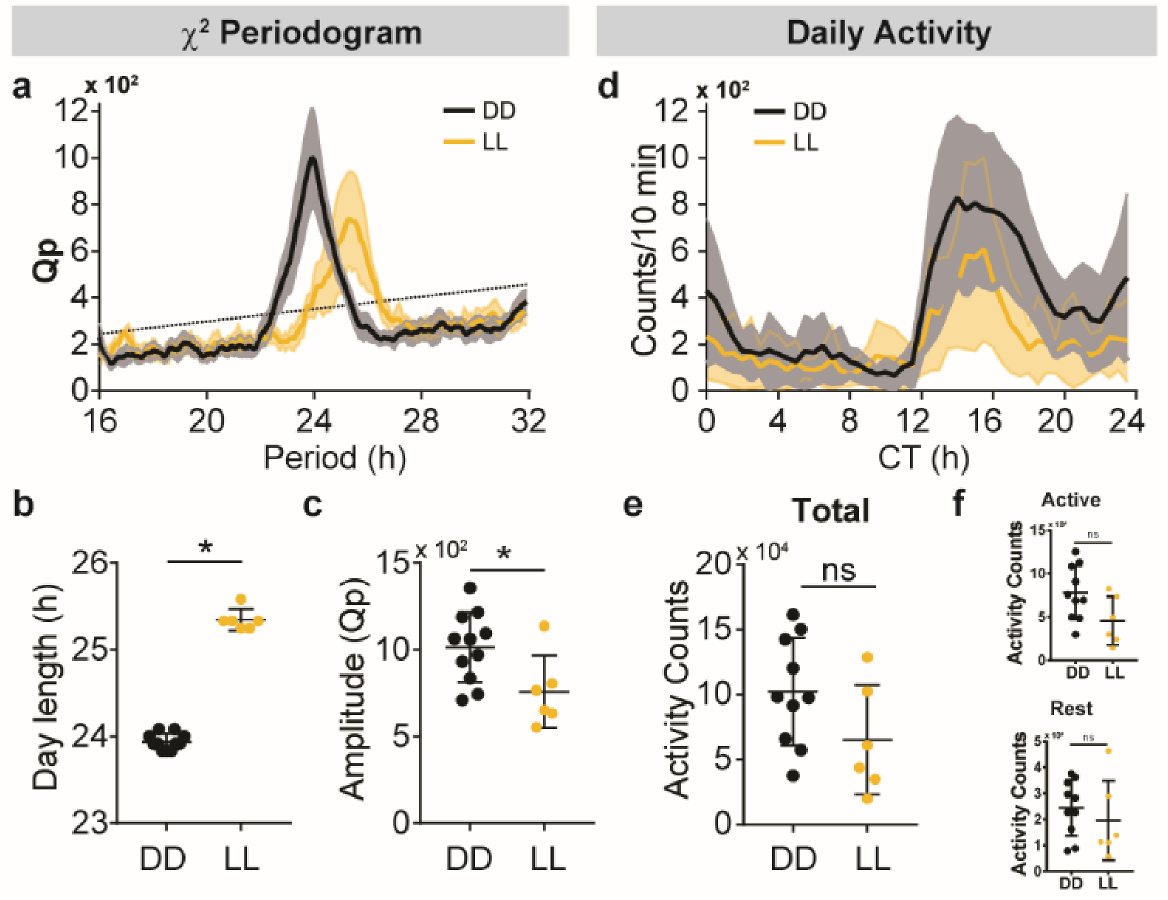

Supplementary Fig. 5. Comparison of mouse cage acitvity in constant dark (DD) and constant light (LL). (a) Average chi-squared periodogram for animals in DD (black, for a-c: n = 10 cages of 2 mice, 1 cage of 1 mouse) and LL (yellow, for a-c: n = 6 cages of 2 mice). Thick lines indicate mean, shading is SEM. Dotted line represents signficant Qp amplitude at different periods. (b) Scatter plot of free running period in DD or LL. Mann Whitney test: $p = 0.026$. (c) Scatter plot of amplitude measures from the χ^2 test. Two-sided Welch's t-test: $p = 0.0325$. (d) Average daily activity profile of animals housed in either DD (for d-f: n = 10 cages of 2 mice) or LL (for d-f: n = 6 cages of 2 mice) for 10 days. The thick lines and shading indicate group average ± SEM. CT is circadian time where CT 12 is activity onset. (e) Scatter plot for total activity counts over a 24h day in DD or LL. (f) Scatter plot for activity counts during the active (top) and rest (bottom) phase in DD or LL. For scatter plots: error bars and center line indicate mean ± SD, individual dots indicate single cage, asterisk indicates $p < 0.05$, ns: not significant. Source data are provided as a Source Data file.

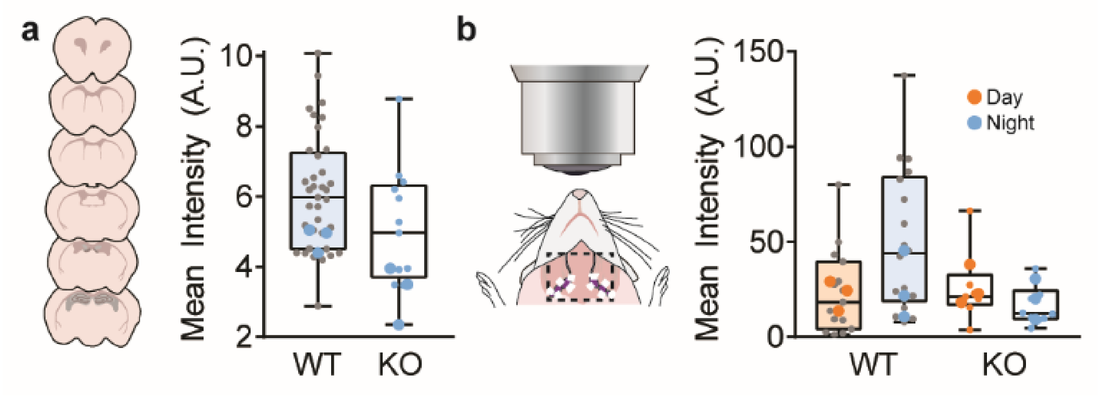

Supplementary Fig. 6. Comparison of WT and AQP4KO glymphatic influx and drainage to the lymph nodes. (a) Schematic of experiment and boxplot of average mean pixel intensity for WT (n = 3 AQP4KO littermates [blue] and 34 C57BL6 mice [gray]) and KO (n = 13 mice) receiving a CM injection during the night. (b) Schematic and boxplot of lymph node intensity in WT (n = 3 AQP4KO littermates per time point [colored] and C57 [gray; day: n = 12 mice, night: n = 15 mice]) compared to KO (day: n = 9 mice, night: n = 10 mice) at 30 min from pump start. Orange: day, blue: night. All boxplots: minima is minimum value, maxima is maximum value, center is median and quartiles shown by box and whiskers, with individual animals shown as colored dots, larger dots indicate animals taken in the same cohort. Source data are provided as a Source Data file.

Supplemental table

Supplementary Table 1. Primers used for RT-qPCR.

Target	Gene ID	Forward Primer Sequence	Reverse Primer Sequence
Aqp4	11829	CTC TGT GCA AAG CTC CTA GTT	GGC AAA GCA GTA TGG GAG AA
Arnt	11863	GGA GTG GAG GAA AGG AGA AAT G	CAC TCA CTC ACT CAC TCA CAA A
Arntl (Bmal1)	11865	CCC GCT GAA CAT CAC AAG TA	GTG GAA GGA ATG TCT GGA GTC
Dag1	13138	GAG CAG TGA GGA CGA TGT TTA	CCC TTC CTC TTC TTG CGA TAG
Dmd	13405	GCT TCT AGT CAT CTG GGC TTA TC	ACA TCT GGA GCC CTA GAC AAT A
Dtna	13527	GTC CAC TTC ACA CCT CTT TAG TT	GAA AGG TCC TCA GGA AGA ATG G
Gja1	14609	GGT GTC TCT CGC TCT GAA TAT C	GTA AGG ATC GCT TCT TCC CTT C
Gjb6	14623	CCG CCT TCA TGT ATG TGT TCT A	TGA AGC AGT CCA CGA GAT TG
Mlc1	170790	CAT CTC TCG GTG ACG TTC TTT	AGA CAC TTG CTG GGA CAT TC
Slc4a4	54403	CCT GGT AGC CTG ACA TGA ATA C	GTG AAA TGA GCC AGG ACT AAG A
Snta1	20648	GAT TGG CTG GCT GAC AGA A	CTG AGG GAG AGA GCA GTA GAA
