# Supplementary material for: Circadian control of brain glymphatic and lymphatic fluid flow
Source: Nat Commun. 2020 Sep 2;11:4411. doi: 10.1038/s41467-020-18115-2 (PMC7468152; doi:10.1038/s41467-020-18115-2)
Supplement: Supplementary file 3 — Description of Additional Supplementary Information [file 41467_2020_18115_MOESM3_ESM.pdf]

Description of Additional Supplementary Files

File Name: Supplementary Movie 1
Description: **Average in vivo CSF tracer influx**. Average macroscopic videos of cisterna magna injected fluorescent cerebrospinal fluid (CSF) tracer influx along the middle cerebral artery during the day (ZT 6-7; n = 6 mice) or night (ZT 18-19, n = 5 mice). Dotted lines indicate region of interest drawn for analysis. Vertical coloured scale bar on the right indicates mean pixel intensity. Time of recording indicated above scale bar. Areas of interest labelled in inset, including the middle cerebral artery (MCA), pineal recess (PR), and the olfactory bulb (Olf.).

File Name: Supplementary Movie 2
Description: **Individual in vivo CSF tracer influx recordings**. 30 minute recordings of fluorescent cerebrospinal fluid (CSF) tracer influx along the middle cerebral artery after injection into the cisterna magna from individual mice during the day (ZT 6-7; n = 6 mice) or night (ZT 18-19; n = 5 mice). Vertical coloured scale bar indicates mean pixel intensity.
