# Supplementary material for: Circadian control of brain glymphatic and lymphatic fluid flow
Source: Nat Commun. 2020 Sep 2;11:4411. doi: 10.1038/s41467-020-18115-2 (PMC7468152; doi:10.1038/s41467-020-18115-2)
Supplement: Supplementary file 6 — Reporting Summary [file 41467_2020_18115_MOESM6_ESM.pdf]

nature research

Corresponding author(s): Maiken Nedergaard

Last updated by author(s): 7/8/2020

Reporting Summary

Nature Research wishes to improve the reproducibility of the work that we publish. This form provides structure for consistency and transparency in reporting. For further information on Nature Research policies, see our Editorial Policies and the Editorial Policy Checklist.

Statistics

For all statistical analyses, confirm that the following items are present in the figure legend, table legend, main text, or Methods section.

n/a	Confirmed	
☐	☒	The exact sample size (n) for each experimental group/condition, given as a discrete number and unit of measurement
☐	☒	A statement on whether measurements were taken from distinct samples or whether the same sample was measured repeatedly
☐	☒	The statistical test(s) used AND whether they are one- or two-sided<br>*Only common tests should be described solely by name; describe more complex techniques in the Methods section.*
☐	☒	A description of all covariates tested
☐	☒	A description of any assumptions or corrections, such as tests of normality and adjustment for multiple comparisons
☐	☒	A full description of the statistical parameters including central tendency (e.g. means) or other basic estimates (e.g. regression coefficient) AND variation (e.g. standard deviation) or associated estimates of uncertainty (e.g. confidence intervals)
☐	☒	For null hypothesis testing, the test statistic (e.g. F, t, r) with confidence intervals, effect sizes, degrees of freedom and P value noted<br>*Give P values as exact values whenever suitable.*
☒	☐	For Bayesian analysis, information on the choice of priors and Markov chain Monte Carlo settings
☒	☐	For hierarchical and complex designs, identification of the appropriate level for tests and full reporting of outcomes
☒	☐	Estimates of effect sizes (e.g. Cohen's d, Pearson's r), indicating how they were calculated

Our web collection on statistics for biologists contains articles on many of the points above.

Software and code

Policy information about availability of computer code

Data collection	For data collection, and transition from Oxymax format to ActogramJ format, CLAX v2.2.15 (animal monitoring software) was used.
Data analysis	The following software and code was used as described in the methods section: ImageJ 1.52i, ActogramJ v1.0 plugin, ITK-SNAP 3.8.0, Advanced Normalization Tools (ANTs) 2.1.0, Python 3.6, Graphpad Prism version 7.0, PASW Statistics 18.

For manuscripts utilizing custom algorithms or software that are central to the research but not yet described in published literature, software must be made available to editors and reviewers. We strongly encourage code deposition in a community repository (e.g. GitHub). See the Nature Research guidelines for submitting code & software for further information.

Data

Policy information about availability of data

All manuscripts must include a data availability statement. This statement should provide the following information, where applicable:

- Accession codes, unique identifiers, or web links for publicly available datasets
- A list of figures that have associated raw data
- A description of any restrictions on data availability

All data needed to evaluate the conclusions in the paper are present in the paper and/or the Supplementary Materials. Additional data available from authors upon request.

Field-specific reporting

Please select the one below that is the best fit for your research. If you are not sure, read the appropriate sections before making your selection.

[x] Life sciences [ ] Behavioural & social sciences [ ] Ecological, evolutionary & environmental sciences

For a reference copy of the document with all sections, see nature.com/documents/nr-reporting-summary-flat.pdf

Life sciences study design

All studies must disclose on these points even when the disclosure is negative.

Sample size	Hablitz et al, 2019 (Sci Advances eaav5447) and Mestre et al. 2018 (eLife 40070) were used to estimate the minimum number of animals required to generate statistically significant datasets, though circadian glymphatic data has never been collected before. Thus, a minimum of 5 animals per group were used.
Data exclusions	No data was excluded from the manuscript.
Replication	Every dataset collected in this manuscript was collected as, at minimum, two separate cohorts of animals to confirm reproducibility of our results.
Randomization	Allocation of all mice into experimental groups was random, though there was an attempt to have equal numbers of male and female mice in each group.
Blinding	In these experiments, all data analysis was blinded. Groups were not assigned until all analyses had been completed, and results were confirmed by at least two separate investigators.

Reporting for specific materials, systems and methods

We require information from authors about some types of materials, experimental systems and methods used in many studies. Here, indicate whether each material, system or method listed is relevant to your study. If you are not sure if a list item applies to your research, read the appropriate section before selecting a response.

Materials & experimental systems

n/a	Involved in the study	
[ ]	[x]	Antibodies
[x]	[ ]	Eukaryotic cell lines
[x]	[ ]	Palaeontology and archaeology
[ ]	[x]	Animals and other organisms
[x]	[ ]	Human research participants
[x]	[ ]	Clinical data
[x]	[ ]	Dual use research of concern

Methods

n/a	Involved in the study	
[x]	[ ]	ChIP-seq
[x]	[ ]	Flow cytometry
[x]	[ ]	MRI-based neuroimaging

Antibodies

Antibodies used	For IHC: Primary antibody used was rabbit anti-AQP4 (Chemicon, AB3594, Lot #: 3213917), secondary antibody used was Alexa 594 donkey anti-rabbit (A21207, Invitrogen), cell nuclei were identified using DAPI (D1306, Invitrogen). For Westerns: Primary Rabbit anti-AQP4 antibody (Chemicon, AB3594, Lot #: 3213917), Donkey anti-Rabbit-HRP antibody (Jackson Immunoresearch, #711-035-152, Lot#59390), Mouse anti-βactin primary antibody (Cell Signaling Technology, #12262S, Lot #: 2), Donkey anti-Mouse-HRP secondary antibody (Jackson Immunoresearch #715-035-150, Lot #: 143140)
Validation	Every new lot of AQP4 primary antibody in our lab is tested with an AQP4 KO mouse control for both western blotting and IHC. The mouse anti-βactin primary antibody was purchased from Cell Technologies and rated for western blotting. According to the Cell Signaling Technologies website it is monoclonal, has reactivity in humans, mice, rats, hamster, monkey, and dog, recognizes endogenous levels of total β-actin protein in multiple cell lines, and was produced by immunizing animals with a synthetic peptide corresponding to amino-terminal residues of human β-actin protein. We confirmed a single band at 45 kDa (Supplementary Figure 4).

Animals and other organisms

Policy information about studies involving animals; ARRIVE guidelines recommended for reporting animal research

Laboratory animals	Male and female C57BL/6 mice (aged 3-5 months, weight between 25 g and 30 g) were acquired from Charles River Laboratories (Wilmington, MA) in equal numbers for each experimental group to control for any potential sex differences. Male and female Aqp4 knockout mice were bred in the University of Rochester vivarium, and backcrossed to C57BL/6 mice for 20+ generations before use. All of the University of Rochester's animal holding rooms are maintained within temperature (18-26 degrees Celsius) and humidity

	ranges (30-70%) described in the ILAR Guide for the Care and Use of Laboratory Animals (1996). Mice were group-housed either in a 12:12 light/dark cycle or under constant light with ad libitum access to food and water.
Wild animals	No wild animals were used in this study.
Field-collected samples	No field-collected samples were used in this study.
Ethics oversight	All experiments were approved by the University of Rochester Medical Center Committee on Animal Resources.

Note that full information on the approval of the study protocol must also be provided in the manuscript.
